# Exercise-Intervened Circulating Extracellular Vesicles Alleviate Oxidative Stress in Cerebral Microvascular Endothelial Cells Under Hypertensive Plus Hypoxic Conditions

**DOI:** 10.3390/antiox14010077

**Published:** 2025-01-10

**Authors:** Smara Sigdel, Shuzhen Chen, Gideon Udoh, Jinju Wang

**Affiliations:** Department of Biomedical Sciences, Joan C. Edwards School of Medicine, Marshall University, Huntington, WV 25755, USA; sigdels@marshall.edu (S.S.); chens@marshall.edu (S.C.); udoh3@marshall.edu (G.U.)

**Keywords:** circulating extracellular vesicles, exercise, cerebral microvascular endothelial cells, hypertension, hypoxia

## Abstract

Our group has recently demonstrated that exercise intervention affects the release and function of bone marrow endothelial progenitor cell-derived extracellular vesicles (EVs) in transgenic hypertensive mice. Whether such an exercise regimen can impact circulating EVs (cEVs) remains unknown. In this study, we investigated the influence of exercise on cEV level and function. Transgenic hypertensive mice (Alb1-Ren) underwent 8-week treadmill exercise (10 m/min for 1 h, 5 days per week). Age- and sex-matched sedentary Alb1-Ren mice served as controls. cEVs were isolated from the blood of exercised and sedentary mice and are denoted as ET-cEV and nET-cEV, respectively. cEVs were labeled to determine their uptake efficiency and pathways. The functions of cEVs were assessed in an Angiotensin II (Ang II) plus hypoxia-injured cerebral microvascular endothelial cell (mBMEC) injury model. Cellular migration ability and oxidative stress were evaluated. We found that treadmill exercise stimulated cEV release, and ET-cEVs were more prone to be internalized by mBMECs. The ET-cEV internalization was mediated by macropinocytosis and endocytosis pathways. Functional studies showed that ET-cEVs can improve the compromised migration capability of mBMECs challenged by Ang II plus hypoxia. Additionally, ET-cEV treatment upregulated the expression of p-Akt/Akt in mBMECs. Compared to nET-cEVs, ET-cEVs significantly reduced ROS overproduction in Ang II plus hypoxia-injured mBMECs, associated with decreased Nox2 expression. All these findings suggest that exercise-intervened cEVs can protect cerebral microvascular endothelial cells against hypertensive and hypoxic injury.

## 1. Introduction

Ischemic stroke is an occlusion in cerebral arteries, resulting in a loss of blood flow, oxygen, and nutrients. Stroke cases have been rising continuously over the years [1,2]. Every forty seconds, someone in the United States suffers from a stroke [3]. It is well recognized that hypertension is one of the major risk factors for ischemic stroke. Despite the benefits of anti-hypertensive therapy, the burden of brain disease, including stroke caused by hypertension, remains substantial [4]. Previous studies have demonstrated that hypertension can impair endothelial function, leading to cerebral supply issues [5,6]. Dysfunction of the endothelium in the brain microvasculature disrupts the balance between vasoconstriction and dilation, promotes leukocyte adhesion, encourages clotting, and prolongs inflammatory responses [7], which leads to exacerbated ischemic stroke damage and worsens outcomes in hypertensive individuals. Indeed, increasing evidence indicates that a cerebrovascular endothelium that is pre-emptively damaged may make an individual more prone to ischemic stroke [8].

Exercise is one of the well-known approaches to prevent and accelerate the recovery of ischemic stroke [9,10]. Exercise interventions have been shown to reduce blood pressure, improve insulin sensitivity, and alleviate pro-inflammatory cytokine levels [11]. Endothelial function may also be improved by exercise intervention, although the type and intensity of exercise could make a difference. It has been shown that moderate-intensity exercise can increase nitric oxide production, which might contribute to endothelial function improvement [12]. We have previously demonstrated that moderate-intensity exercise (10 m/min for 1 h, 5 days per week for 8 weeks) had better effects than low-intensity exercise (5 m/min for 1 h, 5 days per week for 8 weeks) on increasing the number of endothelial progenitor cells in the circulation in C57BL/6J mice [13]. Furthermore, our groups revealed that such an exercise regimen could reduce the cerebral infarct volume and raise microvessel density in middle cerebral artery occlusion-induced stroke in C57BL/6J mice [14]. Although great effort has been made in exercise and cerebrovascular disease research, the effects of and underlying mechanisms responsible for moderate-intensity exercise on the cerebrovasculature in the context of hypertension and ischemia have not been well understood.

Extracellular vesicles (EVs) are membranous particles released by most cell types in response to stimuli or as a part of cell physiology. EVs contain proteins, micro-RNA (miRNA), and lipids, all of which reflect the health status and type of cell they originate from. Yang et al. reported that lipid compositions could affect the size and functions of EVs derived from urine [15]. Increasing evidence suggests that the diverse cargoes of EVs could be carried throughout the blood and even past the blood–brain barrier to be taken up by cerebral cells, making them a crucial method of crosstalk within the body [16]. More recent data reveal that exercise can alter the cargo and functions of EVs. One study has shown that long-term preventative exercise downregulates exosomal miRNAs, such as miR-411-5p, which is associated with cardiovascular death in those with atrial fibrillation [17]. Bei and colleagues reported that a 3-week swimming regimen on C57BL/6 mice increased cEV levels by 1.85-fold. Furthermore, they found that these exercise-intervened cEVs exhibited an anti-apoptotic effect in H_2_O_2_-treated H9C2 cardiomyocytes [18]. Another group found that a 4-week swim exercise did not significantly change the number of cEVs but did change the functions of cEVs in protecting cardiomyocytes against hypoxia/reoxygenation injury. The underlying mechanism was related to the exosomal miR-342-5p. Importantly, the favorable cardioprotective effects and the rise of exosomal miR-342-5p were also observed in exercise-trained human volunteers [19]. Chaturvedi and colleagues showed that the heart and serum exosome concentrations were increased in db/db+ diabetic mouse models following 8 weeks of aerobic exercise. They noted significant increases in miR-29b and miR-455, with the latter being involved in the remodeling of extracellular matrices and fibrosis [20]. We have previously demonstrated that moderate exercise can alter the levels and miRNA profiles of EVs from bone marrow-derived endothelial progenitor cells in C57BL/6J mice [14]. More importantly, these EVs can effectively reduce the extent of cell damage and stroke size following ischemic stroke [14]. Besides the possible effects on EV-miR profiles, exercise intervention has also been shown to alter the protein profiles within EVs, impacting their composition and potential biological effects. Conkright et al. found that an acute heavy resistance back squat exercise changed the surface EV protein density, such as CD63 and vesicle-associated membrane protein, in both men and women subjects [21]. A short time of high-intensity interval training exercise undertaken on a programmable bicycle ergometer has been shown to alter the composition of proteins in cEVs that involve processes such as cellular oxidant detoxification, the regulation of stress, the regulation of exocytosis and vesicle-mediated transport, and response to stimulus in seventeen young men [22]. Aerobic and resistance exercises have been suggested to improve blood lipid profiles, but limited information is available regarding the effects of exercise on EVs’ lipid profiles. Taken together, based on known findings, we speculated that exercise training might affect cEV levels and functions under hypertensive conditions.

In this study, we investigated the effects of moderate-intensity exercise on cEV levels in a hypertensive mouse model and studied whether exercise-intervened cEVs could exhibit favorable effects in protecting cerebral microvascular endothelial cells against hypertension plus hypoxia-induced injury. The combination of exercise and cEV enhancement is crucial to understand in the context of ischemic stroke because it may point to a potential preventative and therapeutic approach to stroke in hypertension.

## 2. Materials and Methods

### 2.1. Animals

Human renin hypertensive transgenic mice (129S/SvEv-Tg; Alb1-Ren; 2Unc/CofJ; the Jackson Laboratory) were used in this study. All experimental mice were inbred and housed in 12 h light/dark cycles at the Marshall University Animal Facility. All mice were given complete free access to normal chow and water. The experimental mice were euthanized at the age of 15–16 weeks old. 

### 2.2. Treadmill Exercise Protocol

The Alb1-Ren mice (male and female; 7–8 weeks old) were randomly assigned to exercise (ET) or sedentary (nET) groups. Exercise protocols were defined in a previous study [14]. All ET mice were adapted to the treadmill (Columbus Instruments, Columbus, OH, USA), running for five days before the beginning of treatment. During day 1 of training, mice ran at 5 m/min for 30 min, and each following day of training, the speed was increased by 1 m/min and the time by 10 min. Following training, the ET mice group underwent 60 min of running at 10 m/min, 5 days/week for 8 weeks.

### 2.3. Cell Culture

The mouse brain microvascular endothelial cells (mBMECs) were purchased from Cell Biologics (Chicago, IL, USA) and cultured according to the manufacturer’s instructions. In brief, mBMECs were cultured in mouse endothelial medium (M1168, Cell Biologics, Chicago, IL, USA) containing growth factor supplements and fetal bovine serum. Cells were washed with 1× phosphate-buffered saline (PBS) every two days and subcultured at 70–80% confluency.

### 2.4. cEV Isolation and Characterization

After an 8-week exercise or sedentary period, the experimental mice were sacrificed. Blood samples were collected from the hearts in syringes containing EDTA anti-coagulant. The samples were centrifuged at 3000× *g* for 20 min to remove platelets. The plasma was then ultracentrifuged at 170,000× *g* for 90 min to collect cEVs. The cEVs isolated from exercised mice were denoted as ET-cEVs, and from sedentary mice were denoted as nET-cEVs. The pellet was resuspended with 100 μL filtered 1× PBS, of which 5 μL was further diluted in 695 μL filtered 1× PBS. This 700 μL sample of diluted cEVs was used for nanoparticle tracking analysis (NTA). Three videos were acquired for the NTA [23]. The camera level was set at nine, and the detection threshold was five. All EV samples were resuspended and diluted in the same way. The formula for EV absolute number in 100 μL of resuspension was calculated as follows: EV (absolute numbers) = NTA concentration (number of particles/mL) × dilution factor (140) × 0.1 (resuspension volume). The formula for EV concentration in plasma was calculated as follows: EV (number of particles/mL) = {NTA concentration (number of particles/mL) × dilution factor (140) × 0.1}/plasma volume (mL). The formula for EV concentration in blood was calculated as follows: EV (number of particles/mL) = {NTA concentration (number of particles/mL) × dilution factor (140) × 0.1}/whole volume (mL). For Western blot analysis, cEV pellets were directly resuspended with 80 μL lysis buffer (Fisher Scientific, Waltham, MA, USA) supplemented with a complete mini protease inhibitor tablet (Roche, Basel, Switzerland).

### 2.5. Internalization Assay of cEVs into mBMECs

To detect the uptake efficiency, cEVs were labeled with PKH26 (Sigma-Aldrich, St. Louis, MO, USA). The labeling protocol was described previously [14]. In brief, following ultracentrifugation, cEV pellets were resuspended with 500 μL PBS, 1 μL PKH26 was added to the suspension, and then the mixture was incubated for 5 min in the dark at room temperature. Then, 500 μL 1% bovine serum albumin (BSA) in PBS was added to stop the reaction. The samples were then ultracentrifuged at 170,000× *g* for 90 min to pellet the cEVs. Then, mBMECs (70–80% confluency) were divided into two groups and incubated with a culture medium supplemented with nET-cEVs or ET-cEVs. The concentration of cEVs was 1 × 10^9^ cEVs/mL, which was chosen based on our previous study [14]. After 24 h of co-incubation, the medium was removed, and cells were rinsed with PBS. The cell nucleus was counterstained with DAPI. The uptake of cEVs by mBMECs was visualized using an inverted fluorescence microscope (Olympus XP70); Boston Industries, Inc., Walpole, MA, USA) equipped with an ACCU-SCOPE Excelis MPX-20RC cooled color microscopy camera (20 Megapixels; NewYork Microscope Company, Hicksville, NY, USA). The images were taken using the same exposure time and light intensity. Four random microscopic images (×20 objectives) in each well represented a group. A blinded investigator analyzed the mean of fluorescence intensities using Image J 1.54f software (NIH, Bethesda, MD, USA). Data were normalized to the fluorescence signal of nET-cEV. The experiment was repeated four times.

### 2.6. cEV Uptake Pathway Determination

We conducted an uptake pathway analysis to determine the internalization pathway of ET-cEVs. In brief, mBMECs were pre-treated with LY294002 (5 μM; inhibitor of macropinocytosis), Filipin (10 μg/mL; inhibitor of lipid rafts-mediated endocytosis), and chlorpromazine (20 μM; inhibitor of clathrin-mediated endocytosis) for 30 min before co-culture experiments. All inhibitors were purchased from Sigma-Aldrich (St. Louis, MO, USA) and were dissolved in DMSO or distilled water according to the manufacturer’s instructions. As DMSO was used as a solvent, the toxic effect of DMSO on cells was evaluated. DMSO did not induce toxicity at the concentrations used, as evidenced by cell viability detection. Then, inhibitors were diluted in a culture medium to achieve the intended work concentrations. The inhibitor concentration was chosen based on previous reports [24]. After 30 min of pre-treatment with the inhibitor, the medium was removed, and cells were washed with 1× PBS. Then, a fresh c μL ture medium (500 μL) supplemented with ET-cEVs (1 × 10^9^ cEVs/mL) was added. A well of cells with no inhibitor treatment served as a control. After 24 h of co-incubation, the medium was removed, and cells were rinsed with PBS. The cell nucleus was counterstained with DAPI. The uptake of cEVs by mBMECs was imaged using an inverted fluorescence microscope (Olympus XP70; Boston Industries, Inc., Walpole, MA, USA) equipped with an ACCU-SCOPE Excelis MPX-20RC cooled color microscopy camera (20 Megapixels; NewYork Microscope Company, Hicksville, NY, USA). The images were taken using the same exposure time and light intensity. Four random microscopic images (20× objective) in each well represented a group. A blinded investigator analyzed the mean of fluorescence intensities using Image J software (NIH, Bethesda, MD, USA). Data were normalized to the fluorescence signal of mBMECs in the control group. The experiment was repeated four times.

### 2.7. Co-Incubation of cEVs with mBMECs Challenged by Ang II Plus Hypoxia

To evaluate the functions of cEVs on hypertension and hypoxia-injured brain endothelial cells, we first challenged the mBMECs with Angiotensin II (Ang II) and hypoxia (1% O_2_ in a Biospherix hypoxia chamber; Biospeherix; BioSpherix, Ltd., Parish, NY, USA) to mimic hypertension and hypoxia injury in ischemic stroke. In brief, before being treated by cEVs, the culture medium of mBMECs in 24-well plates or culture slides was replaced with 500 μL serum and glucose-free medium containing growth factors and 1 uM Ang II (Sigma-Aldrich, St. Louis, MO, USA). Then, the cells were placed in a hypoxia chamber (1% O_2_, 5% CO_2_, and 95% N_2_) for 6 h, followed by 24 h reoxygenation in a standard CO_2_ incubator. During the reoxygenation period, the cells were assigned into different treatment groups and were either untreated or treated with ET-cEVs or nET-cEVs. Cells grown in complete media in the standard incubator were controls. Cells were assayed using the following procedures.

### 2.8. Migration Assay of mBMECs After cEV Treatment

We conducted a scratch assay to determine whether cEVs could rescue the migration capability of mBMECs compromised by Ang II plus hypoxia injury. The mBMECs were exposed to Ang II and hypoxic injury as described and assigned into different treatment groups: no treatment or treated with nET-cEVs or ET-cEVs. Cells cultured with complete culture media in the standard incubator were used as controls. At 0 h, when the cEVs were added to mBMECs, a scratch was made in the middle of each well using a 200 μL pipette tip. Each well was imaged at 0 and 24 h to visualize mBMEC migration. Images were taken using an inverted microscope (Olympus XP70). Image J was used to measure and quantify the scratch area. Data were calculated as Area of changes (%) = (the scratch area in 0 h—the scratch area in 24 h)/the scratch area in 0 h × 100%. Three to four random microscopic images in one well represent a group in each experiment. The experiment was repeated three times. All data were normalized to the fold of the control group.

### 2.9. Reactive Oxygen Species Quantification in mBMECs After cEV Treatment

To determine whether cEVs could impact oxidative stress in mBMECs challenged by Ang II plus hypoxia, we performed Dihydroethidium (DHE, Sigma-Aldrich) staining. In general, mBMECs were exposed to Ang II and hypoxic injury as described and assigned into different treatment groups: no treatment or treated with nET-cEVs or ET-cEVs. Cells cultured with complete culture media in the standard incubator were used as controls. After 24 h of cEV co-culture, 10 uM DHE was added to each well, and the plate was incubated for 70 min. Then, the medium was removed, and cells were rinsed with PBS. The DHE fluorescence signal was imaged with a fluorescent microscope equipped with an ACCU-SCOPE Excelis MPX-20RC cooled color microscopy camera (20 Megapixels). The images were taken using the same exposure time and light intensity. Four random microscopic images (20× objective) in each well represented a group for each experiment. The means of fluorescence intensities were analyzed using Image J 1.54f software (NIH, Bethesda, MD, USA) by a blinded investigator. The experiment was repeated three times.

### 2.10. Western Blot Analysis

Following cEV treatment, cells were collected from each well. The proteins were extracted using lysis buffer supplemented with a complete mini protease inhibitor tablet. A bicinchoninic acid (BCA) assay was performed to quantify protein concentrations of cells and cEVs. Protein lysates were electrophoresed through SDS-PAGE gel before being transferred onto polyvinylidene fluoride (PVDF) membranes, which were blocked with BSA buffer. Primary antibodies CD63 (1:200; Abcam; cat# ab216130), Tsg 101 (1: 200; Abcam; cat# ab30871), Nox2 (1:1000; Abcam; cat# 129068), Akt (1:1000; Cell Signaling Technology; cat# 4691), p-Akt (1:1000; Cell Signaling Technology; cat# 4060), and β-actin (1:4000; Sigma-Aldrich; cat# A5441) were incubated at 4 °C overnight. The next day, membranes were washed and incubated with horseradish peroxidase-conjugated anti-rabbit or anti-mouse IgG (1:40,000; Jackson Immuno Research Lab, West Grove, PA, USA) for two hours at room temperature. Blots were imaged using chemiluminescent solutions in a ChemiDoc imager (Bio-Rad, Hercules, CA, USA) and analyzed using ImageJ 1.54f.

### 2.11. Statistical Analysis

Data are expressed as the mean ± SD. Comparisons between groups were performed using ordinary one-way ANOVA tests and an unpaired *t*-test for cEV quantification. GraphPad Prism 9 was implemented for data analysis. For all measurements, *p* < 0.05 was considered statistically significant.

## 3. Results

### 3.1. Treadmill Exercise Intervention Stimulates cEV Release in Hypertensive Transgenic Mice and Increases the Internalization of cEVs by mBMECs

**Figure 1:**
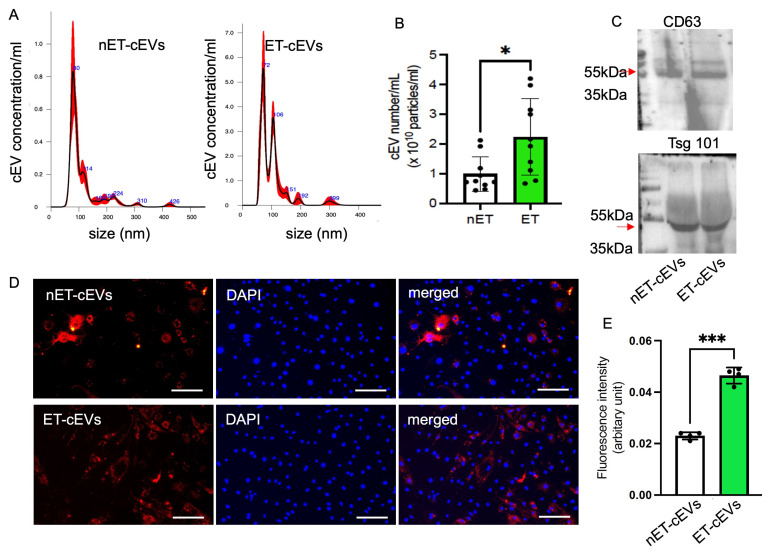
Exercise intervention stimulates cEV release and boosts the incorporation efficiency of cEVs into mBMECs. (**A**) Representative plots showing the size and distribution of nET-cEVs and ET-cEVs. (**B**) Summarized data on the level of the two groups of cEVs. Data are expressed as mean *±* SD. * *p* < 0.05 vs. nET-cEV. n = 10/group. (**C**) Representative Western blot bands showing the expressions of EV-specific markers CD 63 and Tsg 101 (arrow). (**D**) Representative images showing the incorporation of cEVs with mBMECs after 24 h of co-culture. Blue: DAPI; red: PKH-26-labeled cEVs. Scale bar: 100 μm. 200× magnification. (**E**) Summary data of the uptake study. Data are expressed as mean ± SD. *** *p* < 0.01 vs. nET-cEV. n = 4/group.

cEVs were isolated from the plasma of exercised and sedentary hypertensive transgenic mice using the ultracentrifugation method and characterized using NTA and Western blot. According to the NTA analysis of particle size and distribution, the mode size of nET-cEVs was ~80 nm, and ET-cEVs was ~72 nm with another peak of ~106 nm, as shown in a representative plot (Figure 1A). Meanwhile, our data showed that the cEV concentration was approximately 2.24-fold higher in exercised hypertensive mice (2.24 × 10^10^ cEVs/mL of plasma) than in sedentary ones (0.99 × 10^10^ cEVs/mL of plasma) (Figure 1B). According to the Western blot analysis, both groups of cEVs positively expressed EV markers CD63 and Tsg101 (Figure 1C). These findings indicate that treadmill exercise intervention stimulated cEV release in hypertensive transgenic mice.

We recently showed that treadmill exercise improved the internalization of endothelial progenitor cell-derived EVs from exercised hypertensive mice by neuronal cells, but whether such an exercise regimen affects the internalization of cEVs by brain endothelial cells has not been studied. To reveal this, we assessed the effects of exercise on cEV uptake efficiency by brain microvascular ECs. The data showed that more ET-cEVs were internalized into the cytoplasm of mBMECs than that of mBMECs co-cultured with nET-cEVs, as evidenced by a high mean fluorescence intensity in mBMECs treated with ET-cEVs (0.023 vs. 0.046 for nET-cEVs and ET-cEVs, respectively; Figure 1D,E).

### 3.2. ET-cEVs Incorporate into mBMECs Through Endocytosis and Macropinocytosis

**Figure 2:**
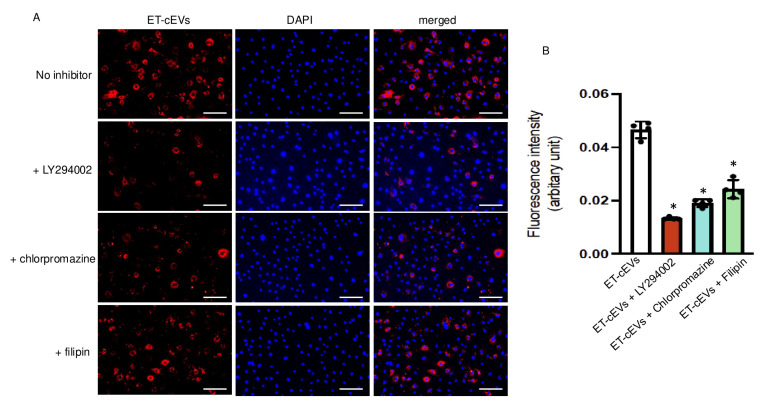
Macropinocytosis and endocytosis modulate the uptake of ET-cEVs into mBMECs. (**A**) Representative image showing the incorporation of ET-cEVs into the cytoplasm of mBMECs pre-treated with or without pathway inhibitors. Blue: DAPI; red: PKH-26-labeled cEVs. Scale bar: 100 μm. 200× magnification. (**B**) Summary data of the uptake mechanism study. Data are expressed as mean ± SD. * *p* < 0.05 vs. ET-cEV only (no inhibitor pre-treatment). n = 4/group.

Several mechanisms, including endocytosis and micropinocytosis, of EV internalization have been proposed. We tested three specific blocking agents to unravel their contribution to cEV internalization by mBMECs. Ly294002, an inhibitor of phosphoinositide 3-kinase, participates in the signaling cascade that stimulates membrane ruffling and closes the macropinosomes during macropinocytosis [25]. Chlorpromazine blocks clathrin-dependent endocytosis by interfering with the association between clathrin and the plasma membrane [26]. Filipin is known to sequestrate cholesterol from the plasma membrane and destabilize caveolae formation. As shown in Figure 2, Ly294002 decreased the internalization of ET-cEVs into mBMECs by 72%, as indicated by the reduced fluorescence intensity in the cytoplasm of mBMECs following 24 h ET-cEV treatment as compared to that in the control group (ET-cEV only) (0.013 vs. 0.046 for ET-cEVs + LY294002 and ET-cEVs, respectively). Chlorpromazine decreased EV uptake by 58% (0.019 vs. 0.046 for ET-cEVs + Chlorpromazine and ET-cEVs, respectively), and Filipin decreased EV uptake by 48% (0.024 vs. 0.046 for ET-cEVs + Filipin and ET-cEVs, respectively). Interestingly, none of the inhibitors can completely block the uptake of labeled vesicles, indicating a combined contribution from both endocytosis and macropinocytosis pathways for EV uptake. Taken together, under the above conditions, Ly294002 exerted the highest inhibitory effect, suggesting that the macropinocytosis pathway may be the main intracellular route for EV intake.

### 3.3. Both nET-cEVs and ET-cEVs Improve the Migration Capability of mBMECs Compromised by Ang II Plus Hypoxia Injury

**Figure 3:**
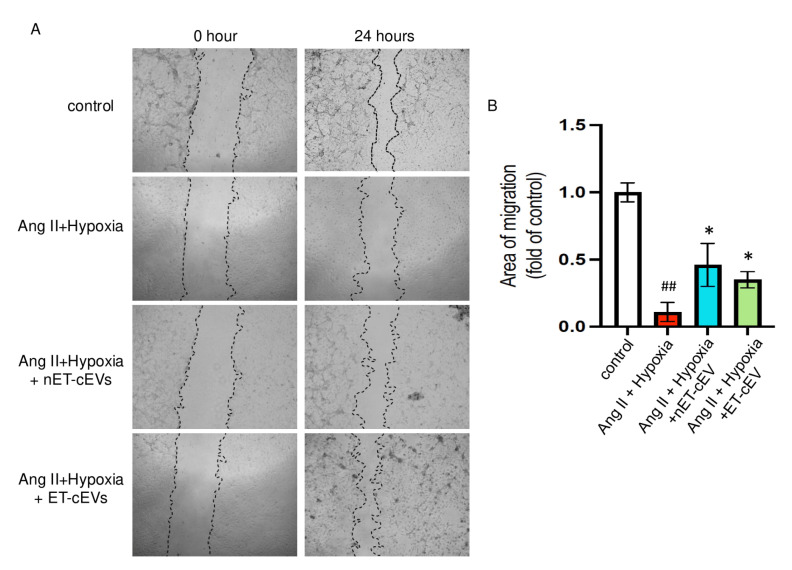
Migration assay of Ang II plus hypoxia-challenged mBMECs treated with cEVs. (**A**) Representative images of mBMEC migration at the baseline (0 h) and 24 h after the cEV treatment; 40× magnification. (**B**) Summary data depict the effects of cEVs on mBMEC migration following Ang II plus hypoxia injury. Data are shown as mean ± SD. ^##^ *p* < 0.01 vs. control; * *p* < 0.05 vs. Ang II + hypoxia. n = 3/group.

A scratch assay was conducted to assess the effects of cEVs on the migration ability of mBMECs. We calculated the area of the scratch in each image of interest. The larger the percentage change from baselines indicates a stronger migration capability of the cells. As expected, the Ang II plus hypoxia challenge significantly impaired the migration ability of mBMECs, as reflected by the smallest percentage change from baseline among all groups, suggesting the successful induction of the cell injury model. As shown in Figure 3, both nET-cEVs and ET-cEVs remarkably improved the migration capacity of mBMECs following Ang II plus hypoxia damage, indicated by a higher percentage change from baseline (at 0 h) compared to the cells without any treatment. Interestingly, there is no significant difference in the migration ability of mBMECs treated by nET-cEVs or ET-cEVs.

### 3.4. ET-cEVs Exhibit Anti-Oxidative Effects in mBMECs Challenged by Ang II Plus Hypoxia

**Figure 4:**
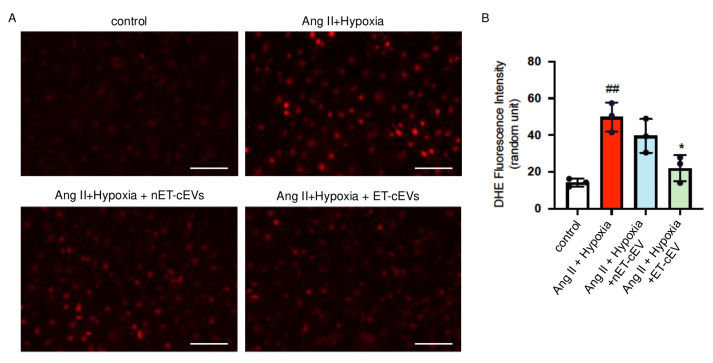
DHE overexpression in Ang II plus hypoxia-challenged mBMECs treated with cEVs. (**A**) Representative image showing DHE expression in hypoxia plus Ang II-injured mBMECs. Scale bar: 100 μm. 200× magnification. (**B**) Summary data of the DHE fluorescence intensity of mBMECs. Data are shown as mean ± SD. ^##^ *p* < 0.01 vs. control; * *p* < 0.05 vs. Ang II + hypoxia. n = 3/group.

DHE staining was conducted to assess ROS overproduction of mBMECs. As shown in Figure 4, compared to the cells cultured in normal conditions, the Ang II plus hypoxia challenge remarkably raised the production of ROS, as revealed by a higher red fluorescence intensity in the cells. The data showed that nET-cEV co-culture alleviated ROS overproduction, but there was no significant difference as compared to that in the non-treated cells. Notably, ET-cEVs significantly decreased ROS overproduction as compared to those treated by nET-cEVs.

### 3.5. ET-cEVs Increase p-Akt/Akt and Decrease Nox2 Expressions in mBMECs Post-Ang II Plus Hypoxia Injury

**Figure 5:**
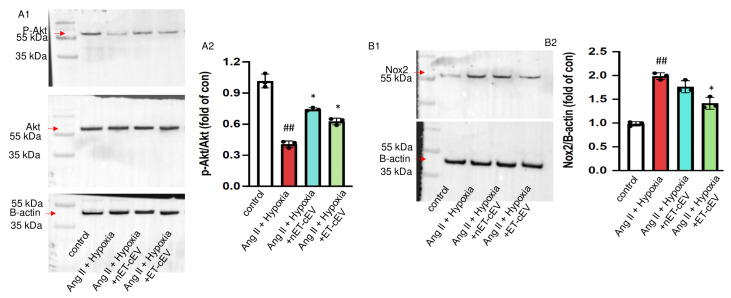
Akt phosphorylation and Nox2 expression in Ang II plus hypoxia-challenged mBMECs treated with cEVs. (**A1**,**A2**) Representative bands and summary data showing the ratio of p-Akt/Akt in mBMECs. (**B1**,**B2**) Representative bands and summary data showing the expression of Nox2 in mBMECs. Data are shown as mean ± SD. ^##^ *p* < 0.01 vs. control; * *p* < 0.05 vs. Ang II + hypoxia. n = 3/group.

Akt, also known as protein kinase B, is expressed in different cell types and plays a central role in various processes, such as migration and proliferation [27,28]. The cellular responses to Akt phosphorylation are executed via signal transduction pathways initiated by the phosphorylation of various Akt substrates [28]. Our data (Figure 5(A1,A2)) revealed that the total Akt expression was unchanged in mBMECs either challenged by Ang II plus hypoxia injury or post-treatment of cEVs. Ang II plus hypoxia challenge significantly decreased Akt activity in mBMECs, as reflected by a decreased ratio of p-Akt/Akt. However, both nET-cEV and ET-cEV treatments robustly restored the activity of Akt, as evidenced by a raised expression of p-Akt/Akt.

It is known that hypoxia is a major source of oxidative stress in hypertension and a potent activator of NADPH oxidases, including Nox2. Therefore, we analyzed the Nox2 level in mBMECs. As shown in Figure 5(B1,B2), Nox2 expression was significantly increased in Ang II and hypoxia-injured mBMECs. The nET-cEV treatment did not remarkably change Nox2 expression, but ET-cEVs significantly decreased Nox2 levels in mBMECs.

## 4. Discussion

In this study, we investigated the effects of exercise intervention on modulating cEV level and function under hypertensive conditions. Our key findings suggest that exercise intervention increased the production of cEVs from hypertensive mice and promoted the uptake of cEVs by mBMECs. The cEVs improved the migration capability of mBMECs, and cEVs from exercised mice further reduced ROS overproduction in Ang II plus hypoxia-challenged mBMECs. The potential mechanism is elucidated by the changes in proteins p-Akt/Akt and Nox2 in mBMECs.

Hypertension impairs endothelial function, which can lead to cerebral supply issues and make the outcomes of ischemic stroke more severe, as represented by increased mortality for hypertensive individuals who experienced an ischemic stroke [1,29]. Fortunately, exercise intervention has been shown to reduce these consequences, but the mechanisms by which this occurs are not clearly understood. Recent evidence indicates that EVs play a pivotal role in maintaining normal cardiovascular function and structure in healthy environments [30,31]. However, in disease conditions such as hypertensive conditions, EVs may decrease nitric oxide release while upregulating ROS production and cellular senescence, eventually leading to vascular lesions [32]. Previous studies showed that aerobic exercise stimulates the release of small EVs into circulation [33,34]. Indeed, exercise-induced EVs emerge as a novel class of exerkines that can exhibit systemic beneficial effects. Our group has previously demonstrated that moderate-intensity exercise stimulated the secretion of EVs from blood-derived stem cells [13]. In this study, we applied the same exercise regimen and studied its effects in hypertensive transgenic mice. Our data showed that moderate-intensity exercise can increase EV production in the blood circulation of hypertensive mice. This trend supports the concept that exercise is an effective type of cellular stimulation regarding EV release. Meanwhile, we found multiple peaks in ET-cEVs according to the NTA analysis. Previous studies suggest that EV size varies depending on their cellular origin and specific cargoes. Yang and colleagues have demonstrated that urinary exosomes of different sizes originated from different types of cells and carry different lipid compositions [15]. The investigation of the specific molecular contents of these ET-cEVs is ongoing. Whether exercise intervention could alter the lipid composition of cEVs in hypertensive conditions and whether such alteration is responsible for the observed effects deserves further study. Additionally, our data showed that exercise intervention alters the uptake efficiency of cEVs. According to our co-culture study, exercise intervention upregulates the incorporation efficiency of cEVs by mBMECs challenged by Ang II and hypoxia, which mimics the injury of hypertension and ischemia in vitro. This finding might represent one of the potential mechanisms underlying the beneficial effects of exercise on ischemic stroke.

In the present study, we also elucidated the potential incorporation mechanism of cEVs by mBMECs. There are numerous mechanisms through which cells may internalize endocytic cargo. Generally, phagocytosis and pinocytosis are the two main subgroups of endocytosis. Phagocytosis involves the internalization of relatively large (>1 μm) particles and is typically restricted to specialized professional phagocytes, such as macrophages and dendritic cells. Pinocytosis is exhibited by all cells and is commonly classified into clathrin-dependent endocytosis, clathrin-independent endocytosis, and macropinocytosis. Several pathways, such as clathrin-mediated endocytosis [35,36] and micropinocytosis, have been reported to mediate EV internalization. Ajikumar and colleagues have demonstrated that endothelial cells take up materials from blood, including EVs, through macropinocytosis [37]. In the present study, we applied pharmaceutical inhibitors to unravel the uptake pathways involved in cEV incorporation. We found that macropinocytosis dominated the uptake pathways of cEVs by mBMECs. Of note, macropinocytosis is not the only pathway by which cEVs enter mBMECs. Endocytosis, to a lesser extent, also contributed to the internalization of cEVs to mBMECs. Future studies will further focus on the molecular mechanisms contributing to the observed internalization process. For instance, lipid compositions that are related to the size of EVs and subsequently influence EV-cell fusion and internalization mechanisms will be studied.

As we know, in an environment devoid of endothelial progenitor cells, endothelial regeneration relies on migration and proliferation, which are critical for cerebral ischemic recovery [38]. Wound scratch and Boyden chamber assays are commonly used to evaluate endothelial cell migration in vitro. In the present study, we conducted the wound scratch assay. We found that cEVs from both exercised and sedentary hypertensive mice substantially restored the migration capability of cerebral microvascular endothelial cells following ischemic hypertensive injury. Notably, the enhanced proliferation capability of cells might also affect their migration ability. Whether the EV cargoes such as miRs, mRNAs, or even DNA fragments could influence the proliferation of the cells has not been studied yet. In the current study, to further understand the corresponding molecular mechanisms of cEVs, we analyzed the expressions of Akt and its phosphorylation in the recipient cells. Akt, the serine-threonine kinase, is present in cells and tissues in both inactive and active (phosphorylated at T308 and S473) states. It is a key molecule in the PI3K/Akt pathway and plays an important role in modulating endothelial function [28]. Akt executes its role through phosphorylation rather than regulating its protein expression. Indeed, accumulating evidence has suggested that the expression level of p-Akt (Ser473) could be upregulated temporarily at the onset of focal cerebral ischemia and downregulated at 24 h after reperfusion, but the expression of Akt was not significantly changed [39]. In the present study, we found that the total expression of Akt was not disturbed by either ischemic hypertensive challenge or cEV treatment; however, the ratio of p-Akt/Akt was significantly decreased in mBMECs challenged by Ang II and hypoxia. Intriguingly, both nET-cEVs and ET-cEVs increased the ratio of p-Akt/Akt. These data are in line with the improved migration capability of mBMECs and are also supported by previous reports showing the positive effects of the Akt pathway in ischemic stroke [27,40]. Such overarching positive effects of cEVs on endothelial injury continue to point to the promise of utilizing cEVs in therapeutic settings for cerebrovascular injury. Previous reports have demonstrated that activation of the Akt pathway might alter the expression of hypoxia-inducible factor 1, which is a downstream target of Akt in cells such as breast cancer cells [41] and mesenchymal stem cells [42]. Whether cEV treatment could modulate the hypoxia-inducible factor 1 level in mBMECs and its potential roles will be investigated in our further studies.

Oxidative stress is a common feature of hypertension and hypoxia conditions. Experimental studies suggest that oxidants are mainly from NOXs in hypertension [43]. Growing evidence indicates that exercise prevents oxidative damage by reducing oxidative stress, an important factor in inflammation and hypertension [44]. However, what remains unclear is exactly how exercise modulates redox status and how it influences proinflammatory processes. In this study, we aimed to clarify this knowledge gap. We speculated that cEVs might be one of the mediators of exercise in decreasing ROS overproduction. Yao et al. have demonstrated that EVs from human mesenchymal stem cells can mitigate oxidative stress in the liver or on umbilical vein endothelial cells [45]. Nie and colleagues reported that skeletal muscle-derived exosomes can regulate the endothelial cell functions of angiogenesis [46]. Our group showed that exercise can regulate the functions of bone marrow endothelial progenitor cell-derived EVs [14]. Endothelial EVs have also been shown to promote the production of endothelial nitric oxide synthase and reduce the effects of oxidative stress [47]. Furthermore, as previously indicated, pathological states such as hypertension can cause cEVs to promote ROS production [32]. In the present study, we found the inability of cEVs from sedentary hypertensive mice to reduce ROS overproduction following hypertensive hypoxia injury, which supports previous findings. Meanwhile, it was abundantly clear to us that cEVs from exercised hypertensive mice can dramatically reduce ROS overproduction in mBMECs following ischemic hypertensive injury, indicating that exercise intervention can regulate cEV function in hypertensive conditions. Our data also revealed that ET-cEVs treatment significantly decreased Nox2 expression, while nET-cEVs did not in mBMECs challenged by Ang II and hypoxia. This is in line with the data showing that ET-cEV treatment reduced ROS overexpression. It is also supported by a recent study demonstrating that exercise-intervened endothelial progenitor cell-derived exosomes can protect neurons by improving mitochondrial function [48]. Knowing that cEVs from exercised conditions are more effective in reducing ROS, which suggests that their contents may change in response to aerobic activity, we strove to understand the mechanism behind the effects of cEVs on the endothelium at a deeper level. We will further analyze the contents of cEVs, such as miRs, and lipid compositions, and illustrate the underlying mechanism. Skeletal muscle is one of the major tissues that release EVs under exercise intervention. Whether skeletal muscle-derived EVs play a role in the effects elicited by ET-cEVs is unknown and will also be studied.

There are some limitations in this study: (1) We only investigated the effects of exercise intervention in cEVs in hypertensive transgenic mice. Whether this exercise regimen could affect the levels and functions of cEVs from normotensive mice needs to be investigated in future studies. (2) Subpopulations of cEVs, such as skeletal muscle-derived EVs, brain endothelial cell-derived EVs, bone marrow-stem cell-derived EVs, etc., that might play major roles in the observed effects require further investigation.

## 5. Conclusions

In conclusion, our data show that moderate-intensity exercise can stimulate cEV release in hypertensive conditions, and cEVs, specifically those from exercised mice, can protect mBMECs against hypertensive and hypoxia injury. The underlying mechanisms are ascribed to the anti-oxidative and pro-angiogenic effects of cEVs. Combining our findings regarding how ET-EVs can modulate oxidative stress pathways along with previous research showing that size-dependent EV lipid compositions [15] and oxidized lipids significantly contribute to endothelial dysfunction and atherosclerosis [46] provides important insight into how exercise might protect against cerebrovascular diseases such as stroke in the context of hypertension through EV-mediated mechanisms. Our findings solidify the importance of exercise intervention in maximizing the beneficial contributions of cEVs to alleviate the damage in cerebrovascular vessels caused by hypertension and hypoxia and have suggested that exercise-intervened cEVs might be a new therapeutic target for ischemic stroke in the context of hypertension.

## Data Availability

The datasets generated during the current study are available from the corresponding author upon reasonable request.
